# Preventing collateral damage

**DOI:** 10.7554/eLife.74319

**Published:** 2021-11-02

**Authors:** Harry Quon, Fred Bunz

**Affiliations:** 1 Department of Radiation Oncology and Molecular Radiation Sciences, Johns Hopkins University School of Medicine Baltimore United States

**Keywords:** dietary nitrate, cancer, radiotherapy, miniature pig, Other

## Abstract

In pigs, nitrate supplements can protect salivary glands from the damage caused by radiation therapy to the head and neck.

**Related research article** Feng X, Wu Z, Xu J, Xu Y, Zhao B, Pang B, Qu X, Hu L, Hu L, Fan Z, Jin L, Xia D, Chang S, Wang J, Zhang C, Wang S. 2021. Dietary nitrate supplementation prevents radiotherapy-induced xerostomia. *eLife* **10**:e70710. doi: 10.7554/eLife.70710

Most patients with head and neck cancer will receive radiation therapy in order to kill or shrink their tumor (Alterio et al., 2019). During treatment, physicians try to minimize damage to surrounding, healthy tissues, but off-target doses often harm and kill the sensitive ‘serous acinar cells’ in the salivary parotid gland (Figure 1A). As a result, many patients go on to produce less saliva and develop a persistent dry mouth, also known as xerostomia. This is not a benign condition: people may experience loss of taste, difficulty chewing, swallowing or speaking and, in the long term, tooth and gum decay that can lead to malnutrition (Jensen et al., 2019).

**Figure 1.**
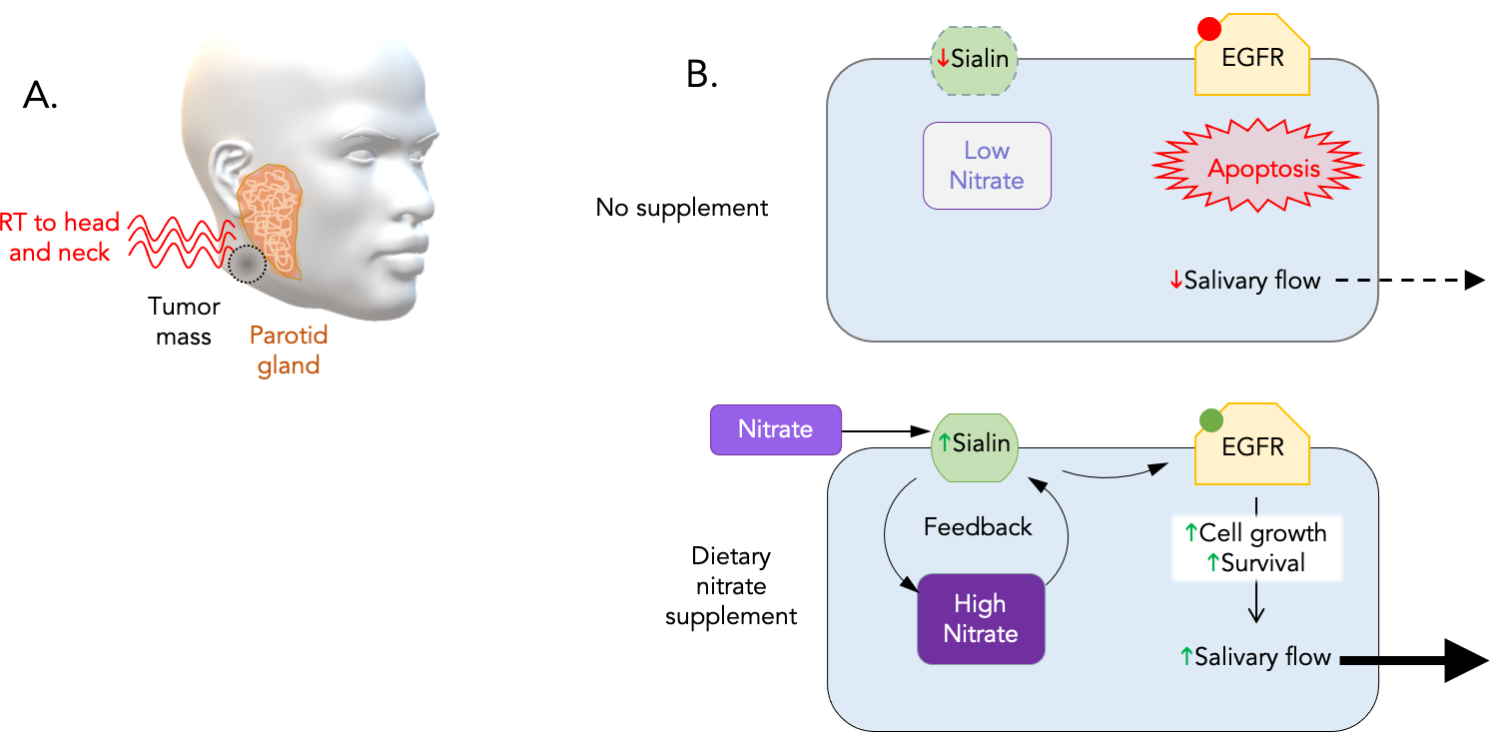
Dietary nitrate initiates a positive feedback that stimulates the growth and survival of serous acinar cells. (**A**) The parotid gland (orange), which is a major source of saliva, often becomes damaged when head and neck cancer patients receive radiation therapy (red waves) to kill or shrink their tumors (dark grey). (**B**) To explore ways to protect this sensitive gland, Feng et al. examined how the nutrient nitrate affected miniature pigs exposed to radiation. This revealed that the animals that do not receive additional nitrate before treatment experience a rapid decrease in salivary flow. Irradiated human parotid cells (top) had decreased levels of sialin (green), a protein that transports nitrates into serous acinar cells (blue rectangle) which produce saliva. As a result, the level of nitrate inside the cell drops, and EGFR (yellow) – one of the components of the EGFR-AKT-MAPK signaling pathway – remains in an inactive state (red dot). This causes the cells to die (apoptosis), perhaps explaining the decline in the flow of saliva in irradiated pigs and humans. Adding inorganic nitrate to the cells before treatment (bottom) increases sialin and therefore nitrate levels inside the cell. In turn, nitrate could increase the production of sialin, creating a positive feedback loop that allows the cell to maintain relatively stable amounts of sialin after radiation. This triggers the activation of EGFR (green dot), which stimulates the cell to grow and prevents programmed cell death.

Few interventions exist to stop this side effect from emerging, aside from technical refinements that limit the exposure of the glands to radiation (Mercadante et al., 2021). Now, in eLife, Songlin Wang and colleagues at Capital Medical University – including Xiaoyu Feng and Zhifang Wu as joint first authors – report a remarkably simple measure that may protect salivary glands during radiation therapy (Feng et al., 2021).

In the body, these glands are an important component of the nitrate cycle, taking up about 25% of the inorganic nitrate present in the blood, concentrating it and then secreting it into the saliva (Lundberg et al., 2018). This nutrient, abundant in leafy greens and many fruits, was once reviled for potentially causing cancer but it is now viewed as a normal component of a healthy diet. It can even help to boost the regeneration of certain heart cells (Lundberg et al., 2018; Marino et al., 2021).

Feng et al. used miniature pigs – whose salivary glands are structurally similar to those of humans – to investigate whether nitrate could help protect against xerostomia after radiation therapy. Animals that were fed daily doses of inorganic nitrate before treatment did not experience a sharp drop in saliva production, and they recovered 80% of their salivary flow within two years.

These benefits were both dose- and time- dependent: higher amounts of supplementary nitrate led to better salivary gland function, but administering the nutrients for the first time two months after treatment yielded minimal results. In the laboratory, adding inorganic nitrate to cells derived from human parotid tissues revealed a similar radioprotective effect. Taken together, these results strongly support supplementing patient’s diets with nitrate to prevent xerostomia.

Exactly how nitrate can protect cells against radiation is not fully understood, but it may involve sialin, a transport protein that helps to usher the nutrient inside serous acinar cells. Feng et al. showed that radiation caused the levels of sialin to rapidly fall. Adding nitrate before treatment, however, boosted the level of sialin, and therefore the amount of the nutrient inside cells. Additional experiments suggest that nitrate then increases the production of sialin, creating a positive feedback loop that activates the EGFR-AKT-MAPK pathway (Figure 1B). This biochemical circuit is known to stimulate cell growth and block programmed cell death (Seshacharyulu et al., 2012). The production of sialin in response to nitrate appeared to be the critical trigger for EGFR activation, which may explain why supplementation was only effective if administered before radiation therapy.

Extrapolating from animal and cell-based models to humans is always uncertain (Mak et al., 2014). Perhaps the most exciting aspect of the work by Feng et al. is that its main conclusion is easy to test, through randomized clinical trials that monitor salivary function (and potentially nitrate levels) before, during and after radiation therapy. This will ultimately help to determine whether nitrate supplementation could offer a low-tech solution to a high-tech problem. If the stunning results presented by Feng et al. translate to humans, this approach may have a major impact on cancer patients experiencing xerostomia.
